# Aflatoxin B_1_ induces infertility, fetal deformities, and potential therapies

**DOI:** 10.1515/med-2024-0907

**Published:** 2024-01-26

**Authors:** Sullibie Francis, Nii Korley Kortei, Marian Sackey, Seidu A. Richard

**Affiliations:** Department of Obstetrics and Gynecology, Ho Teaching Hospital, P.O. Box MA-374, Ho, Ghana; Department of Nutrition and Dietetics, School of Allied Health Sciences, University of Health and Allied Sciences, Ho, Ghana; Department of Pharmacy, Ho Teaching Hospital, P.O. Box MA-374, Ho, Ghana; Department of Medicine, Princefield University, P. O. Box MA128, Ho, Ghana

**Keywords:** AFB_1_, infertility, testis, ovary, malformations, markers

## Abstract

Aflatoxin B_1_ (AFB_1_) is a subsidiary poisonous metabolite, archetypally spawned by *Aspergillus flavus* and *A. parasiticus,* which are often isolated in warm or tropical countries across the world. AFB_1_ is capable of disrupting the functioning of several reproductive endocrine glands by interrupting the enzymes and their substrates that are liable for the synthesis of various hormones in both males and females. In men, AFB_1_ is capable of hindering testicular development, testicular degeneration, and reduces reproductive capabilities. In women, a direct antagonistic interaction of AFB_1_ with steroid hormone receptors influencing gonadal hormone production of estrogen and progesterone was responsible for AFB_1_-associated infertility. AFB_1_ is potentially teratogenic and is responsible for the development of malformation in humans and animals. Soft-tissue anomalies such as internal hydrocephalus, microphthalmia, cardiac defects, augmented liver lobes, reproductive changes, immune modifications, behavioral changes and predisposition of animals and humans to neoplasm development are AFB_1_-associated anomalies. Substances such as esculin, selenium, gynandra extract, vitamins C and E, oltipraz, and CDDO-Im are potential therapies for AFB_1_. Thus, this review elucidates the pivotal pathogenic roles of AFB_1_ in infertility, fetal deformities, and potential therapies because AFB_1_ toxicity is a key problem globally.

## 1 Introduction

Aflatoxin B_1_ (AFB_1_) is a subsidiary poisonous metabolite, archetypally spawned by *Aspergillus flavus* as well as *A. parasiticus* [1,2,3,4]. These species of molds are routinely isolated in warm or tropical countries across the world [1,5]. This type of fungi is extremely pathogenic to animals and humans [6]. The name B, “blue,” was adopted based on the blue light of ultraviolet (UV) radiation [6]. AFB_1_ has been isolated in various types of foods, such as cereals (corn, wheat, millet, and rice), oleaginous plants (soya, sunflower, and cotton), spices (chilly, black pepper and coriander), nut fruits (pistachio, coconut, almond, etc.), dried fruits and vegetables, milk, and other meat products [7,6,8,9,10,11,12].

It is worth noting that approximately 4.5 billion of the world’s population is exposed to aflatoxins [9,10]. Underdeveloped and poor countries in Africa and Asia have registered acute toxicity of AFB_1_ in humans, and chronic toxicity has been observed in people who consume food with minute concentrations of AFB_1_ contained in organisms [5,8–10]. Thus, the American Food and Drug Administration and the European Union have legally regulated normal or abnormal concentrations of aflatoxins in animal as well as human foods [13,14]. Notably, AFB_1_-contaminated food is often ingested via mouth into the gastrointestinal system through which the toxins gain access to targeted organs via the blood stream.

AFB_1_ has been implicated as a pathogenic factor in child underweight, neurologic injuries, hypoimmunity, cancer such as hepatocellular as well as high mortality [15,16]. Globally, human fertility is diminishing, a state that cannot be ascribed exclusively to an upsurge in contraception [9,17]. It was observed that in about 30% of infertility, pathology originated from men alone, and in another 20%, the pathology often originated from both men and women [18]. Thus, the male factor is accountable for infertility in about 50% of patients who present at the clinic [18]. However, there is currently no data suggesting that AFB_1_ affects males than females or vice versa in the clinic.

Also, regression in semen quality has a substantial negative influence on male fertility and is thus a public health concern. Also, follicle growth and atresia in the ovaries and fallopian tubes, resulting in severe infertility, are also major public health concerns. Environmental factors have been implicated as a major cause of regression in semen quality, follicle growth, and atresia of the ovaries and fallopian tubes, although the precise triggers are still a matter of debate [9,19]. Thus, this review elucidates the pivotal pathogenic roles of AFB_1_ in infertility, fetal deformities, and potential therapies.

The “Boolean logic” was used to search for articles on the subject matter in PubMed and PubMed central as well as Google scholar with search terms like AFB_1_ and associated diseases in humans and animals, AFB_1_ and infertility in humans and animals, AFB_1_ and reproductive hormones, AFB1 and deformities, and potential therapies to AFB_1_. Studies involving both humans and animals were included. Also, findings from both clinical research and basic research were critically reviewed.

## 2 AFB_1_ induces diseases in humans and animals

Interestingly, AFB_1_ has been linked to hepatotoxic, nephrotoxic, genotoxic, mutagenic, and immunotoxic [20,21]. Also, AFB_1_ triggered an immunosuppressive effect, resulting in a decrease in the natural and acquired resistance to diseases when ingested at very low levels [22,23]. Notably, AFB_1_ has been detected in the blood during the acute phase of illness after exposure, as well as in the liver of affected children [24–27]. Nevertheless, use of aspirin or phenothiazines was also assumed to be associated with the etiology [28]. Furthermore, AFB_1_ has been isolated in the blood of pregnant women, in neonatal umbilical cord blood, and in breast milk in African countries, with cyclical disparities [25,29]. Also, the highest concentrations of AFB_1_ ever observed in human tissue and fluids were detected in the umbilical cord blood at birth [24], which signify that AFB1 crosses the placenta and could trigger fetal deformities.

Intriguingly, AFB_1_ has been implicated as the etiological factor in encephalopathy and fatty degeneration of viscera, analogous to Reye syndrome in countries with a hot and humid climate [24,30]. Also, patients presented with pale, fatty liver, enlarged kidneys, and severe cerebral edema [24]. Notably, aflatoxicol was detected in the serum, liver, urine, and stools of children with kwashiorkor and marasmic kwashiorkor compared to marasmus and control children, where this metabolite was not detectable [24]. Moreover, as high as 80% of AFB_1_ was detected in the serum and 46% in the urine of infants with kwashiorkor and marasmus [31].

Remarkably, children with kwashiorkor and marasmic kwashiorkor who were fed an AFB_1_-free diet, had a very slow elimination of AFB_1_ during clinical investigations [32]. Also, AFB_1_ was isolated in the brain and lungs of children who had died from kwashiorkor, as well as children who had died from diverse diseases [24,27,33]. Furthermore, AFB_1_ was detected in the lungs of all children who suffered from pneumonia, regardless of the presence of kwashiorkor [24]. This may be due to a decrease in the eliminatory ability of the lungs in pulmonary diseases and/or due to contact via the respiratory route [24].

Interestingly, a correlation study on the existence of AFB_1_ in the serum and urine of children and the prognosis of acute lower respiratory infection did not yield any association [34]. Also, AFB_1_ was detected in the lungs of some textile workers and farmers who died from pulmonary interstitial fibrosis [24,35]. Furthermore, acute human aflatoxicosis was associated with liver failure and gastrointestinal bleeding in Southeast Asia and Africa [24]. Notably, AFB_1_ was connected to a specific AGG to AGT amino acid transversion mutation at codon 249 of the p53 gene in human hepatocellular carcinoma, specifying mechanistic evidence to a causal link between exposure and disease [36].

Interestingly, AFB_1_ was capable of triggering acute hepatic injury, resulting in the elevation of serum enzymes, such as lactate dehydrogenase, aspartate aminotransferase, glutamate dehydrogenase, gamma-glutamyltransferase, and alkaline phosphatase [37]. Furthermore, bilirubin, which indicates liver damage, and other biochemical factors like proteinuria, ketonuria, glycosuria, and hematuria were elevated in AFB_1_-triggered acute live injury [37]. Moreover, about 72% of kids had perceptible concentrations of AFB_1_-lys in their plasma at 24 months of age. However, no relation was established between the low AFB_1_ ingestions and growth impairment [38].

Moreover, the focal severe distraction of the renal cortex, which was not only limited to the renal tubules but also stretched into the renal corpuscles, resulting in a wide gap in the urinary spaces, has been associated with AFB_1_ [24]. It is worth noting that augmented collagen deposition and focal mononuclear cell infiltration were detected in renal system after AFB_1_ administration [39]. Furthermore, it was observed that AFB_1_ triggered focal necrosis and degeneration, predominantly at the renal tubules [40]. Also, AFB_1_ was capable of triggering lymphocytic infiltration, necrosis, and steatosis in the liver of ducklings [41].

Intriguingly, AFB_1_ was capable of triggering aberrations in the mitochondrial DNA of brain cells, resulting in malfunctioning oxidative phosphorylation [42,43]. The defective oxidative injury resulted in disruptions in key cellular macromolecules, such as DNA, lipids, and proteins [42,43]. Also, cellular fatty acids are freely oxidized by reactive oxygen species (ROS) induced by AFB_1_ to generate lipid peroxyl radicals, which in turn proliferate into malondialdehyde (MDA), and the resultant MDA interrelates with cellular DNA to form DNA–MDA, which influences the generation of energy in the brain [42,43]. Notably, AFB_1_ has been implicated to influence ovarian secretory cells via the hypothalamus–hypophysis–ovary axis [44].

Studies on the effect of AFB_1_ and key reproductive hormones mediated by brain regions are insufficient. Also, it is worth noting that via the above organs and associated diseases, AFB_1_ may migrate to productive organs to induce infertility. Thus, correlation studies between the above diseases and infertility are warranted.

## 3 Isolation of AFB_1_ in reproductive organs

Notably, mice fed with AFB_1_ diet showed histological alterations like germ cell loss in their testis, while aflatoxicosis was associated with reduction in sperm production and augmented sperm abnormalities in male mice [45–47]. Also, AFB_1_ exposure in the testis of male mouse triggered a reduction in sperm concentration as well as motility and an upsurge in aberrations leading to reduced fertility in the mice [46]. Notably, AFB_1_ associated spermatogenesis, with almost total absence of spermatids engaging in spermiogenesis, accompanied by the loss of immature germ cells leading to decreased sperm count, was detected histologically [46]. Interestingly, these pathological changes triggered by AFB_1_ in the testis were observed in the Leydig cells mice. Also, major histopathological changes in the epididymis were associated with AFB_1_ in the testis of mice [48]. Thus, a direct toxicity of AFB_1_ to the spermatogenic compartment is the key mechanism of action of AFB_1_ in the stimulation of abnormal sperms. Remarkably, AFB_1_ was associated with substantial upsurge in oxidative stress markers and reduction in anti-oxidant enzymes in the testicles of rats [49].

Notably, isolated AFB_1_ in the ovaries triggered damage in the ovary and increased the risk of ovarian disease; moreover, zearalenone has a dual effect on ovarian toxicity induced by AFB_1_ [50]. In the rat ovaries, the detected AFB_1_ inhibited follicle growth and atresia in the ovaries resulting in severe infertility [51]. Interestingly, the detected AFB_1_ triggered multiple signaling pathways in the ovary and induced oxidative stress, affecting genes associated with sterol, amino acid, and lipid synthesis during transcriptomic analysis [50]. Interestingly, the detected AFB_1_ in the ovaries inhibited the growth of oocytes, reduced the ovary size and weight, reduced oestradiol-17β concentration, and increased progesterone concentration in blood after AFB_1_ administration in female rats [44,52]. It is well established that mycotoxins are capable of crossing the placental barrier and have already been isolated in human umbilical cord samples [53,54]. Interestingly, injection of a single dose of AFB_1_ in the pre-implantation period affected uterine growth and triggered failure of fetal development [55].

## 4 Biotransformation of AFB_1_ in the body

Biotransformation or metabolism is the means by which a chemical substance is altered or transformed from one chemical state to another via successions of enzymatic or chemical response(s) inside the body and subsequently excretion of the byproducts or metabolites mostly via renal excretion [56–58]. In toxicology, biotransformation is very crucial in the defense mechanism via the excretion of toxic xenobiotics and body wastes in which they are transformed into less detrimental and polar substances that are easily excreted [56–58].

The process of elimination, which often encompasses metabolism and excretion of chemical substances from the body, comprises two principal phases [56,59]. Phase I involves the metabolism of chemical substances via the addition of small polar groups comprising of both positive and negative charges to xenobiotics of aflatoxins via the process of acetylation, oxidation, reduction, and hydrolysis, which render it harmless [56,59]. Phase I is mainly intermediated via the cytochrome P450 (CYP450) enzyme systems [56,59].

In contrast, phase II metabolism often encompasses glucuronide, glutathione, sulfate, and amino acid conjugation reactions [57,60]. The whole metabolic process permits Phase I products to “fit” into Phase II enzyme cascades where they are capable of combining with another substance to yield a polar or water-soluble substance that can certainly be excreted via the kidneys [57,59]. Nevertheless, in some instances, some of the chemical substances may be transformed into reactive or harmful products [56]. Microsomal enzymes metabolize AFB_1_ to distinctive metabolites via hydroxylation, hydration, demethylation, and epoxidation in the liver [61] and may migrate to the reproductive organs to induce infertility.

Interestingly, cytochrome P450-mediated metabolism is the primary focus of the biotransformation of AFB_1_ [56]. Nevertheless, AFB_1_ bioactivation via prostaglandin H synthase and lipoxygenase may be more essential than P450-catalyzed bioactivation in certain experimental systems [62,63]. Notably, AFB_1_ is bioactivated via epoxidation of the terminal furan ring double bond, producing an electrophilic intermediate, AFB_1_-8,9-epoxide, a stereoisomer that consists of both the *exo* and the *endo* configurations [64–66].

Interestingly, AFB_1_-*exo*-epoxide was proficient in alkylating nucleic acids and proteins, while the AFB_1_-*endo*-epoxide was a very weak mutagenic [64]. Furthermore, AFB_1_-*exo*-epoxide was easily crystallized in high quantities and steady in aprotic non-nucleophilic solvents [62,67]. Moreover, AFB_1_-exo-epoxide was capable of reacting with an extreme concentration of DNA, resulting in the formation of 98% of AFB_1_-DNA adducts although it had a half-life of approximately 1 s in an aqueous buffer [62,67].

AFB_1_ was also capable of stimulating 8-hydroxy-20-deoxyguanosine (8-OHdG) configuration in livers of rats and ducks during *in vivo* treatment [68–70]. Additionally, AFB_1_ triggered the elevation of 8-OHdG levels following the treatment of cultured woodchuck hepatocytes [62,69]. It was established that the most commonly detected mutation stimulated by AFB_1_ was DNA alkylation via AFB_1_-exo-epoxide followed by the AFB_1_-N7-Gua formation leading to G-T transversions [62,69].

Interestingly, AFB_1_-triggered ROS formation involves metabolism via cytochrome P450 to form AFB_1_-exo-epoxide and/or the hydroxylated metabolite AFM_1_ and requires both the participation of iron-catalyzed reactions and Kupffer cells in the rat livers *in vivo* and in rat hepatocytes [62,71,72]. Also, oxidative damage was capable of triggering AFB_1_ toxicity, resulting in a reduction in the anti-oxidant-capable parameters related to apoptosis [73,74]. Remarkably, AFB_1_ augmented apoptotic cells in spleen, broilers jejunum, and bursa fabricius [75–77]. Also, AFB_1_ augmented the secretion of fundamental liver apoptotic markers like Bcl-2-associated X protein (Bax), caspase-3, and p53 as well as reduced the secretion of key anti-apoptotic markers like B-cell lymphoma 2 (Bcl-2) [73]. Moreover, MDA was markedly elevated while superoxide dismutase (SOD) and the total anti-oxidant capacity (T-AOC) were much lower in AFB_1_-treated mice [16].

Notably, the MDA tissue content often reflects the degree of oxidative damage because it is a peroxide generated via free radicals [78]. Markedly, AFB_1_ stimulated oxidative stress, which was observed via the peroxidation of lipids and MDA in the serum [16]. Furthermore, AFB_1_ triggered the expression of free radicals, particularly superoxide anions, in kidney tissue where numerous T-AOC factors, such as SOD in the serum, were conscripted into the tissue, leading to downregulation of T-AOC and SOD in serum [16]. Thus, the effects of AFB_1_ on these factors are coherent with their stimulation of oxidative reactions in the mice [16].

It is worth noting that SOD is a typical anti-oxidant enzyme in diverse organisms, which translates superoxide anion radicals to hydrogen peroxide and safeguards organisms from oxidative injury. However, T-AOC is marker of total antioxidative activity and it reflects the activity of all the anti-oxidants in an organism [79]. Interestingly, proline dehydrogenase (ProDH) mRNA and protein were expressively elevated in AFB_1_-treated mice, and cell apoptosis in kidney tissues was similarly expressively stimulated and was associated with varying secretions of Bcl-2, Bax, and caspase-3 [16].

Moreover, proline levels were low in kidney tissue obtained from AFB_1_-treated mice, which was possibly due to ProDH upregulation [16]. Furthermore, ProDH siRNA was utilized to determine whether ProDH was a direct target of AFB_1_ [16]. In this experiment, it was established that the secretion of pyrroline-5-carboxylate synthase (P5CS), pyrroline-5-carboxylate reductase (P5CR), and proapoptotic factors were not distinctive in AFB_1_-treated and small interfering RNA (siRNA)-treated cells, and in cells that were treated with ProDH siRNA alone [16]. Thus, downstream apoptotic factors, such as Bcl-2, Bax, and caspase-3, were influenced by ProDH siRNA treatment [16].

It was established that AFB_1_ was capable of changing the metabolism of tryptophan in the brain, which decreases the levels of serotonin [80,81]. It was also observed that recurrent exposure to AFB_1_ resulted in the reduction of striatal dopamine and serotonin levels by 37% and 29%, respectively, signifying that AFB_1_ influenced dopaminergic and serotoninergic pathways, probably via selective triggering of the translation of tyrosine to biogenic catecholamine neurotransmitters [80,82]. Furthermore, acute AFB_1_ exposure reduced brain acetylcholinesterase, while the chronic exposure augmented adenohypophyseal acetylcholinesterase [80,83]. Thus, this indicates that neurotransmitters are influenced by AFB_1_, which leads to hormonal imbalance and maybe infertility. Further studies are needed in this direction.

## 5 AFB_1_ and reproductive hormones

AFB_1_ was capable of disrupting the functioning of several endocrine glands by interrupting the enzymes and their substrates that are liable for the synthesis of diverse hormones. Notably, AFB_1_ and generated ROS were capable of causing cancers in endocrine glands, such as pituitary gland, granulosa cell tumors of the ovary as well as adenomas and adenocarcinomas of the adrenal gland, ovaries, testes, kidneys, thyroid gland, parathyroid glands, and pancreas [84,85]. Interestingly, AFB_1_ is capable of inducing cancer as result of its capacity to generate several altered forms of DNA adducts. Thus, AFB_1_ was capable of adversely influencing the reproductive capacity of male and female animals [84,85].

**Figure 1:**
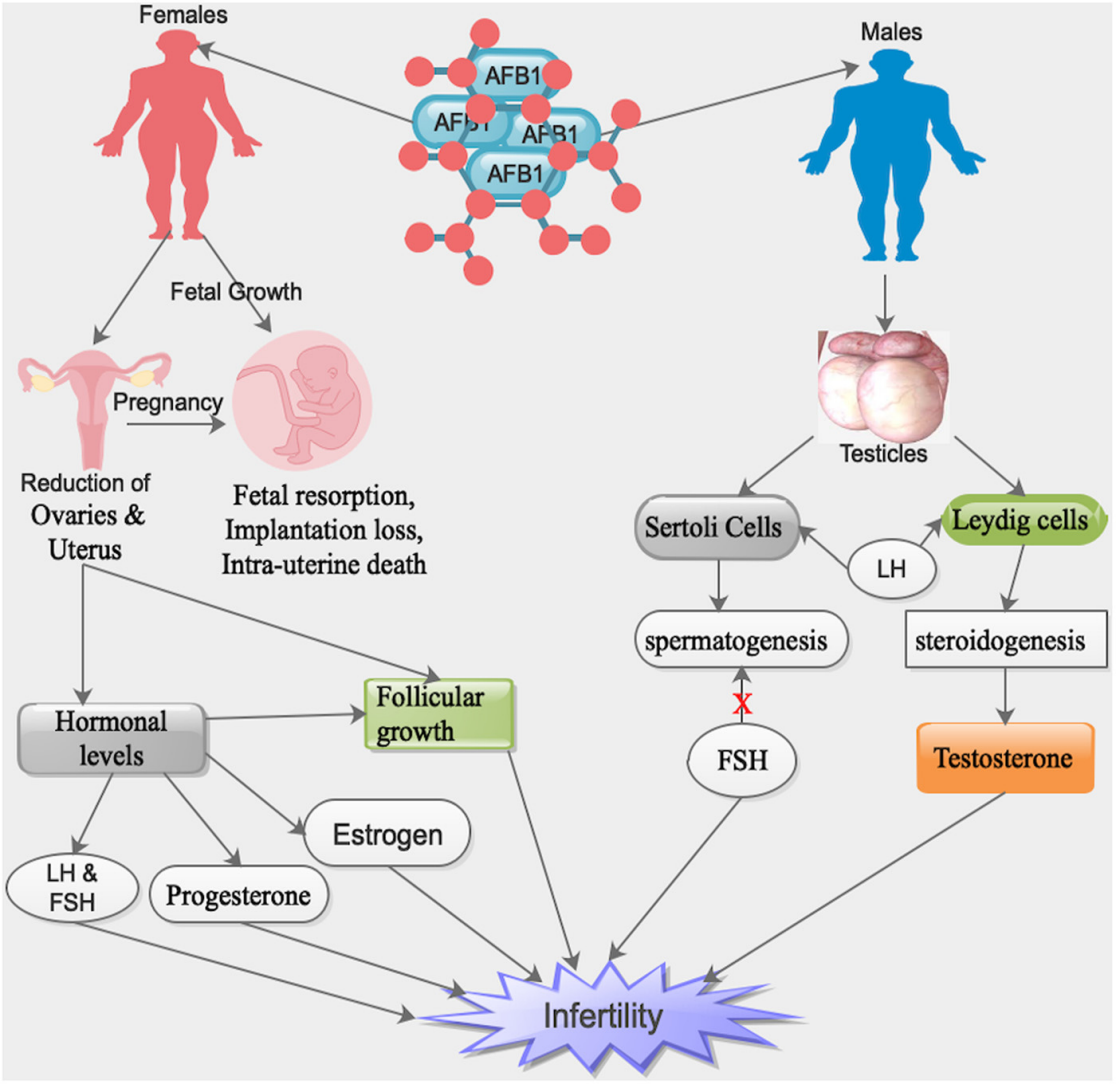
Influence of the ingested AFB1 by various ways in both male and female reproductive organs, resulting in infertility and/or fetal instability during pregnancy. X = inhibitory. Note: human symbols denote both humans/animals.

Notably, AFB_1_ was capable of inducing a reduction in the sizes of ovaries and uterus as well as augmentation in the rates of fetal resorption, implantation loss, and intra-uterine death in AFB_1_-exposed female rats (Figure 1) [86]. Furthermore, AFB_1_ was capable of influencing sexual maturation, follicular growth and maturation, hormonal levels, pregnancy, and growth of fetus (Figure 1) [86]. Interestingly, in male mammals, 17β-estradiol is produced by Leydig cells as a result of a feedback reaction to luteinizing hormone (LH) and by Sertoli cells as a result of a feedback reaction to follicle-stimulating hormone (FSH) (Table 1) [87].

**Table 1:** Various types of hormones influenced by AFB_1_ and their mechanisms

Hormone	Effect of AFB1 on the hormone	Mechanism of action	References
Estrogen	Downregulation	Direct antagonistic interaction of AFB_1_ with steroid hormone receptors influencing the gonadal hormone production of estrogen, as a result of structural similarity of AFB_1_ and steroid hormones	[50,86,104]
Luteinizing hormone (LH)	Downregulation	Diminished serum testosterone concentration triggered a decreased sensitivity of Leydig cells to LH and/or direct blockade of testosterone production in rats exposed to AFB_1_	[87,91,92,94]
Follicle-stimulating hormone (FSH)	Upregulation	Decreased levels of circulatory testosterone with higher concentrations of FSH during AFB_1_ exposure	[86,87,91,92,95]
Testosterone	Downregulation	Upsurge in cholesterol levels in the testis of AFB_1_-treated mice could be a result of inadequate utilization of cholesterol or impaired steroidogenesis	[87,90–92,108]
Progesterone	Upregulation	AFB_1_ in the placenta was capable of triggering an upsurge in progesterone synthesis	[50,97,104]
		Direct antagonistic interaction of AFB_1_ with steroid hormone receptors influencing gonadal hormone production of progesterone, as a result of structural similarity of AFB1 and steroid hormones	
Hepatic alphafetoprotein (AFP)	Upregulation	AFB_1_ adversely influences AFP production, which in turn inhibited the gonadal function resulting in a reduction in the concentrations of hormonal promoters	[98]

Also, in male mammals, testosterone biosynthesis transpires in the Leydig cells of the testis as a result of a feedback reaction to LH produced by the pituitary gland (Table 1) [88]. Cholesterol is a substrate for steroidogenesis and it is transported into mitochondria via steroidogenic acute regulatory (StAR) proteins [89]. Cholesterol is converted into pregnenolone by cytochrome P450, a side-chain-cleaving enzyme in the mitochondria [89]. Subsequently, pregnenolone is transported into the smooth endoplasmic reticulum where it is converted into testosterone [88].

Remarkably, diminished steroidogenesis is a key phenomenon in AFB_1_-mediated reproductive toxicity [90,91]. Further studies on the exact mechanism via which AFB_1_-mediated reproductive toxicity occurs during steroidogenesis are warranted. Interestingly, an upsurge in cholesterol levels in the testis of AFB_1_-treated mice was observed [91]. Remarkably, the upsurge in cholesterol levels in the testis of AFB_1_-treated mice could be as result of inadequate utilization of cholesterol or impaired steroidogenesis (Table 1) [91].

Moreover, a reduction in serum testosterone concentrations in rats following exposure to AFB_1_ was observed (Table 1) [92,93]. The diminished serum testosterone concentration was probably due to a decreased sensitivity of Leydig cells to LH and/or direct blockade of testosterone production in rats exposed to AFB_1_ (Figure 1) [92,93]. Markedly, a substantial upsurge in serum concentrations of FSH with reduced testosterone levels in AFB_1_-treated rats was detected compared to controls (Table 1) [87]. It is worth noting that a decreased concentration of circulatory testosterone with higher concentrations of FSH and LH signifies an intact pituitary–testicular axis in AFB_1_-treated rats (Table 1) [88,92,93].

Intriguingly, FSH was capable of stimulating Sertoli and Leydig cells, resulting in the regulation of spermatogenesis and steroidogenesis, respectively (Figure 1) [94,95]. It is worth noting that an upsurge in the serum FSH concentration signifies an inhibition of spermatogenesis in AFB_1_-treated rats and suggests germ cell loss or impairment of Sertoli cells, resulting in impaired feedback regulation of FSH secretion (Figure 1 and Table 1) [88,96]. Furthermore, LH was also capable of stimulating Sertoli and Leydig cells, resulting in the regulation of spermatogenesis and steroidogenesis, respectively (Figure 1 and Table 1) [88,95].

Also, it was observed that chronic exposure to AFB_1_ was capable of triggering endocrine disruption in the human foetoplacental component because it was capable of influencing the secretion of aromatase enzymes, such as P450 or CYP enzymes (Figure 1) [97]. Thus, AFB_1_ was a hypothetical endocrine disruptor, which was capable of influencing steroid ovarian hormones levels, either directly or indirectly [97]. Furthermore, AFB_1_ was capable of augmenting the secretion of CYP19A1 in human placenta cells [97,98].

Furthermore, AFB_1_ was capable of influencing key genes in endocrine regulation in placental cells after being metabolized into aflatoxicol [97]. Also, AFB_1_ influenced placental steroid hormone production, metabolism, and conjugating enzymes, which triggered abnormalities in the foetoplacental hormonal homeostasis [98]. Moreover, CYPs have been implicated in steroid hormones production and the upsurge of the secretions of these enzymes by AFB_1_ in the placenta was capable of triggering an upsurge in progesterone synthesis (Figure 1) [97].

It was established that modifications in oestradiol-17β and/or progesterone levels during the luteal phase and/or the orchestrated oestrus had unfavorable influences like shortened cycles, lower fertility, negative influence on follicle maturation, ovulation, or the existence and/or the signs of the oestrus cycle on succeeding reproductive lifecycle of the animals (Figure 1) [44]. Moreover, blood oestradiol-17β and progesterone were expressively lower and higher, respectively, in rats exposed to AFB_1_ (Figure 1 and Table 1) [44]. Thus, AFB_1_ had a direct influence on ovarian secretory cells or on the hypothalamus–hypophysis–ovary axis [44].

Interestingly, after exposing AFB_1_ to male rats for 48 days, it was observed that the levels of blood serum LH, testosterone, and oestradiol-17β were expressively lower in the group of rats exposed to the highest dose of AFB_1_ (Figure 1 and Table 1) [87]. Thus, AFB_1_ had direct influence on testes secretory cells or on the hypothalamus–hypophysis–testis axis [87]. Notably, AFB_1_ adversely influenced hepatic alphafetoprotein (AFP) production, which is identified to inhibit gonadal function, resulting in the reduction in the concentrations of hormonal promoters above (Table 1) [99].

## 6 AFB_1_-associated infertility in humans

The disruptive abilities of AFB_1_ have been observed in the reproductive system in both male and female human beings after consumption of AFB_1_-contaminated foods [37]. Testicular damage has been reported in infertile men as a result of early accumulation of AFB_1_ in human systems [9]. Patients who are exposed to chronic AFB_1_ develop a lower percentage of sperm morphology even with the very low cut-off value for sperm morphology, as recommended in the new edition of the World Health Organization (WHO) semen analysis [9].

AFB_1_ was capable of hindering testicular development, testicular degeneration, reduced reproductive capabilities, morphological regressive modifications in the testis, and blockage of Leydig cell function in men (Figure 1) [9,100]. AFB_1_ was isolated in the blood and semen of infertile men [47]. Interestingly, isolated AFB_1_ was 25% in the semen of infertile patients as compared to 2.1% in controls [9]. Furthermore, detection of abnormal semen parameters, such as severe decrease in the sperm count, decreased motility, high percentage of abnormal morphology, and high viscosity in the semen of the infertile group compared to that in the fertile group as well as the WHO reference values for normal semen parameters (Figure 1) [9].

Notably, a similar study observed a prevalence rate of AFB_1_ in 40% of infertile men compared to 8% in fertile men [101]. Remarkably, 50% of the infertile men with high AFB_1_ semen levels also exhibited abnormalities in semen parameters. Also, AFB_1_ was capable of damaging chromosomes, genes, and forming aflatoxin–DNA complex, which triggered key anomalies in human sperms [102,103]. Furthermore, AFB_1_ was capable of generating ROS in the form of free radicals, which triggered the anomalies in human sperms [9].

ROS were able to influence macromolecules like protein, DNA, lipid of the sperm and testicular tissues, resulting in cellular/tissue damage after exhaustion of natural anti-oxidants [9]. However, selenium and/or vitamin E supplements in cases of idiopathic male infertility were capable of augmenting the quality of semen and boosting the production and protective effects on sperm motility [104]. Also, circulating ROS generated by aflatoxins in cases of idiopathic male infertility were neutralized by these above potent anti-oxidants [9].

Interestingly, a substantial increase in the mean ovarian volume in infertile females and a substantial reduction in the mean follicular size was observed (Figure 1) [105]. It is worth noting that two distinct adverse activities have been implicated as causes of AFB_1_-related female fertility [105]. These two actions are an indirect influence facilitated by AFB1-stimulated hypovitaminosis A and a direct antagonistic interaction of AFB_1_ with steroid hormone receptors influencing gonadal hormone production of estrogen and progesterone as a result of structural similarity of AFB_1_ and steroid hormones (Figure 1 and Table 1) [105].

## 7 AFB_1_-associated infertility in animals

AFB_1_ has been implicated in the disruption of reproductive systems in both male and female animals after ingestion of AFB_1_-contaminated foods [37]. Experimental studies in animals revealed that certain AFB_1_ was capable of influencing the reproductive abilities of both sexes resulting in anomalous sperm, low sperm count, sterility, and/or affect hormone activity leading to infertility [9,106]. Furthermore, AFB_1_ was capable of reducing motility of sperms obtained from ejaculation or epididymis (Figure 1) [9,106]. Thus, AFB_1_ is capable of decreasing the number of primary spermatocytes, spermatids, and the morphology of sperm cells produced (Figure 1) [107,108].

Notably, the concentration of plasma testosterone and 5α-dihydrotestosterone (5α-DHT) as well as absolute and relative testicular weights of experimental male animals exposed to AFB_1_ remained low in all age groups, and a tardiness in the onset of sexual maturation during aflatoxicosis (Table 1) [109]. Furthermore, AFB_1_ was capable of triggering pathological modifications, such as degeneration and necrosis of epithelial cells of sperm tubules and decrease in the number of sperms (Figure 1) [110]. Also, AFB_1_ was capable of decreasing the semen volume, testicular weight, spermatocrit, plasma testosterone, and a decrease in the egg output in poultry (Figure 1) [37,111].

Intriguingly, continuous feeding of male goats with diets containing AFB_1_ triggered testicular degeneration (Figure 1) [112]. Also, AFB_1_ was capable of delaying the physiological, behavioral sexual maturation, and testicular development in Japanese quail (Figure 1) [113]. Furthermore, AFB_1_ was capable of reducing the semen volume and testis weight, which resulted in the interference of the germinal epithelium in mature male white Leghorn chicks [114].

Moreover, degenerating alterations of diverse intensity in the germinal epithelium of the seminiferous tubules led to devastating dystrophic changes in the spermatogenic epithelium alongside edematous alterations in the interstitial tissue in adult male rats exposed to AFB_1_ diet for prolonged periods were observed (Figure 1) [90,115]. Similarly, degeneration in the epithelium lining of seminiferous tubules and congestion of testicular blood vessels with intertubular edema in the rats exposed to AFB_1_ was observed (Figure 1) [116].

Furthermore, coagulative necrosis of the whole epithelium lining of several seminiferous tubules, which were transformed into homogenous eosinophilic debris in their lumina, was also observed [116]. Also, AFB_1_ was capable of decreasing the number of Leydig cells, the height of seminiferous tubules, the number and the index of sertoli cells, the diameter of caput epididymis, and lumen caput epididymis (Figure 1) [110]. Moreover, the number of spermatogenesis, spermatocytes, and spermatids was also decreased [110].

In female experimental animals, AFB_1_ was capable of triggering pathological modifications in the form of coagulative necrosis, particularly in the growing and mature follicles, resulting in a reduction in the number and size of graffian and growing follicles with augmented number of atretic follicles and a slight portion of degenerative alterations (Figure 1) [37,105]. Also, in laboratory and domestic female animals, AFB_1_ was capable of triggering a decrease in ovarian and uterine sizes, augmented fetal resorption, implantation loss, and intra-uterine death in female rats exposed to AFB1 (Figure 1) [37,105].

Interestingly, AFB_1_ wields all types of teratogenic effects on growing and non-growing follicles, which result in the reduction of ovulatory follicles in rats (Figure 1) [9,51]. Furthermore, infertility parameters like disturbances of estrus cyclicity, blockade of lordosis, and decrease in conception rates and litter sizes were detected in rats exposed to AFB_1_ [117]. Also, mature domestic fowls exposed to AFB_1_ exhibited follicular atresia during histopathological examinations of their ovaries, which resulted in the cessation of egg production during the whole feeding period [111].

## 8 AFB1 and fetal deformities

AFB_1_ presents a potential hazard to animal and human health in view of their teratogenicity [118,119]. Notably, AFB_1_ could be accountable for the development of malformation in humans [120–122]. Several detrimental consequences like low birth weight, small litters, fetal death and resorption, bone and visceral deformities, reproductive changes, impact on immune capacity, and behavioral changes, and predisposition to neoplasm development are related to exposure to AFB_1_ during the prenatal period [5]. Specifically, fetal anomalies are observed when the pregnant animals are exposed to AFB_1_ via gavage or intramuscularly [123].

Notably, reduction in weight and absolute size of the viscera, decreased size of the heart sinusoid capillaries, as well as ventricular lumen, liver, and kidneys containing vacuoles as well as congestion, atrophy, glomerular degeneration, and disorganization of hepatocyte were detected in fetus exposed to AFB_1_ during the prenatal period [119,124]. Also, anomalies in organs like thymus presenting lymphoid depletion and decrease in epithelial differentiation, moderate degeneration of the testicles with atrophy, and the decrease of germ cells of seminiferous tubules were detected in fetus exposed to AFB_1_ during the prenatal period [125].

Interestingly, at the cellular level, AFB_1_ was capable of augmenting the numbers of both apoptotic cells and mitoses in the periportal regions; the nuclei of some cells were distended; hyperchromatic, and pleomorphic with a coarse chromatin pattern with cytoplasm’s that were markedly melanocytosic [119]. Notably, AFB_1_ was capable of triggering chromosome aberrations in bone marrow of rats [126–128]. Remarkably, mutagenicity of AFB_1_ resulted in the formation of covalent N7 guanine adducts, which interrupted DNA replication, leading to anomalies in the chromosomes [129,130]. Also, AFB_1_ was capable of stimulating chromosomal aberrations, such as centromeric attenuations, chromatid breaks, chromatid gaps, end-to-end associations, chromosomal fusions, ring chromosomes, dicentric chromosomes, fragments, deletions, centric fusions, stickiness, and hypoployploidy in the bone marrow cells of rats [119,131,132].

**Figure 2:**
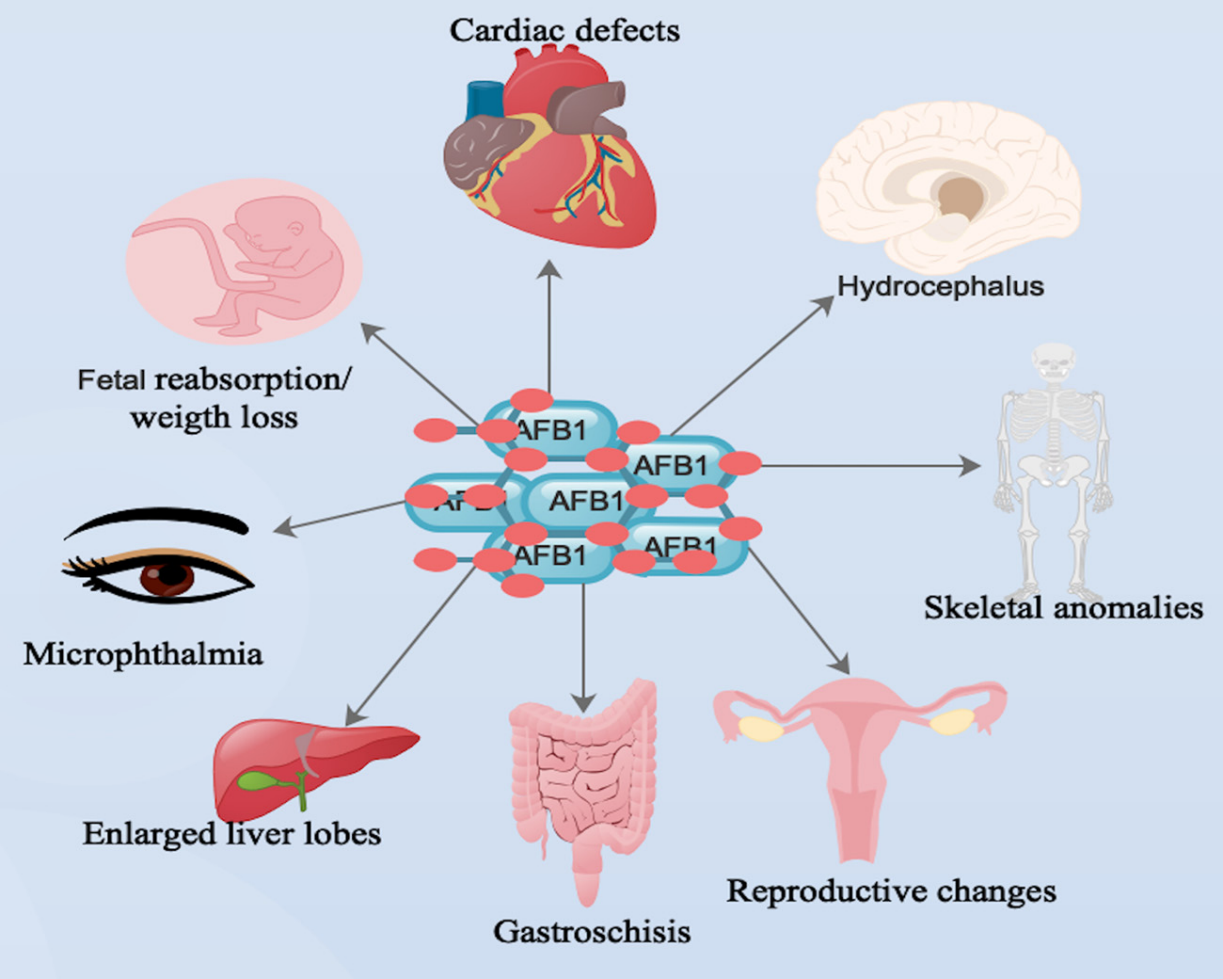
Main body organs that AFB1 was capable of triggering deformities during prenatal exposes. Note: human symbols denote both humans/animals.

Markedly, AFB_1_ was capable of causing skeletal anomalies with incomplete ossification of skull bones and failure of ossification of small bones (Figure 2) [133,134]. These skeletal defects were seen mostly in the ribs and soft-tissue anomalies [133,134]. Furthermore, AFB_1_-associated deformities are a result of the formation of phenotypic abnormalities due to delayed metamorphosis [119]. Also, AFB_1_ triggered some errors during transcription of developmental genes and defects in homoeotic genes, which influenced the final ailment of imaginal discs [135].

Interestingly, daily consumption of a mixture of AFB_1_ and AFG_1_ by pregnant white rats from the 8th to the 12th day of gestation triggered a decrease in the number of implantation sites and fetal weight and augmented the reabsorption of fetuses (Figure 2) [136,137] as well as the absence of one or more coccygeal vertebrae and, in some cases, compaction of the vertebral column [119]. Furthermore, AFB_1_-associated bone defects were usually linked to ossification failures, alterations in bone size, and shape, and the absence or modification of some bone issues (Figure 2) [123].

Intriguingly, AFB_1_ was capable of influencing the transcription of genes linked to bone development, affecting activities like intramembrane mineralization and endochondral ossification [55,123,134,138]. Also, animals exposed to AFB_1_ during embryonic development were more prone to minor malformations, like those affecting bone fates was detected (Figure 2) [119]. These minor malformations were mostly limb defects, such as the absence of some metacarpal and metatarsal bones and some phalanges [119]. Furthermore, AFB_1_-triggered mineralization-associated defects were linked to the direct effect of AFB_1_ on the osteoblasts, osteoclasts, and periosteal cells [119].

Notably, impairment in the mineralization of osteoid tissues, such as bone matrix, affected bone maturation and prevention of periosteal new bone formation parameters like endochondral and intramembranous ossification [119,134]. Also, exencephaly and gastroschisis were observed in mice fetuses exposed to AFB_1_ [139]. Furthermore, soft-tissue anomalies, such as internal hydrocephalus, microphthalmia, cardiac defects, and augmented liver lobes were observed (Figure 2) [140]. Also, multilobulation of the liver in one fetus exposed to a higher dose of AFB_1_ was detected [140].

Notably, localization of ^14^C-labeled AFB_1_ by the pigment layer of fetal eye, liver, and heart, and continuous exposure of AFB_1_ during the organogenesis period was responsible for the manifestation of distinctive abnormalities of these organs (Figure 2) [141]. Also, AFB_1_ was associated with reproductive changes, immune modifications, behavioral changes, and predisposition of animals and humans to neoplasm development (Figure 2) [5,123].

## 9 Potential therapies for AFB_1_

Detoxification techniques generally used to destroy AFB_1_ are physical and chemical methods on contaminated foods [142]. Physical approaches, such as heat and gamma rays are the most typical techniques for neutralizing AFB_1,_ while chemicals such as acids, bases, oxidizing agents, and reducing agents are the most typical techniques used to destroy or extinguish AFB1 on contaminated foods (Table 2) [142]. The use of plant extracts to degrade AFB_1_ and the inoculation of bacterial strains in food substrates are two main biotechnological techniques used to reduce the AFB_1_ levels in contaminated foods (Table 2) [142].

**Table 2:** Agent/chemical/drug and the modes of actions used in the treatment of contaminated food, exposed to animals and/or human beings

Agent/chemical/crug	Treatment (mode) food/animal/human	Mode of action of agent/chemical/drug	Reference
Physical (heat and gamma rays)	Contaminated food	Neutralization of AFB1	[141]
Chemical (acids, bases, oxidizing agents, and reducing agents)	Contaminated food	Destruction or extinguish AFB1	[141]
Biotechnological (plant extracts and bacterial strains)	Contaminated food	Degradation of AFB1	[141]
Novasil clay minerals	Animal and humans (oral)	Absorption of AFB1 *in vitro*	[141]
Phyllanthus amarus	Humans (oral)	Augmentation lipid peroxidation, leading to downregulation of AFB1 in the liver	[143,144]
Black tea	Humans (oral)	Augmentation lipid peroxidation, leading to downregulation of AFB1 in the liver	[143,144]
Gynandra extract	Animals and humans (oral)	Anti-oxidant	[39,40,145]
Esculin	Animals and humans (oral)	Anti-oxidant	[39,40,145]
Selenium	Animals and humans (oral)	Anti-oxidant	[39,40,72,76,145]
Ascorbic acid (vitamin C)	Animals and humans (oral)	Anti-oxidant	[146]
Vitamin E	Animals and humans (oral)	Anti-oxidant	[38,90,148]
Oltipraz	Animals (oral)	Reduction of hepatic AFB1-derived DNA adducts	[150–152]
CDDO-Im	Animals and humans (oral)	Multifunctional agent with anti-inflammatory, antiproliferative, apoptotic, and cytoprotective activities	[156–159]

However, novasil clay minerals have been proven to possess high affinity and combine well with AFB_1_ in the gastrointestinal tract [142]. Novasil clay minerals were capable of absorbing AFB_1_ *in vitro* in both animal models and human studies (Table 2) [142]. They were able to decrease the bioavailability of blood toxins, and their usage in humans did not influence the utilization of vitamins and trace elements in the body during clinical trials [142]. Natural plant products are synthetic antibacterial agents with biodegradability, biosafety, effectiveness, and regenerability capabilities [143].

Interestingly, the anti-oxidant influence of *Phyllanthus amarus* herbal extracts and black tea were capable of augmenting lipid peroxidation, which is often downregulated by AFB_1_ in the liver (Table 2) [144,145]. Also, studies have shown that substances, such as esculin, selenium, and gynandra extract, which have anti-oxidant functions, are capable of modifying AFB_1_-stimulated oxidative stress, resulting in the relieving of the resultant histological anomalies (Table 2) [40,41,146].

Intriguingly, standard anti-oxidants like ascorbic acid (vitamin C) were capable of neutralizing the harmful effects of AFB_1_ on most hematological, biochemical, and enzymatic parameters (Table 2) [147]. Additionally, selenium had protective effect in the spleen and liver against AFB_1_-stimulated toxicity by blocking oxidative stress and associated extreme apoptosis (Table 2) [73,77].

Moreover, selenium was capable of decreasing mitochondrial swelling and mitochondrial DNA mutations in ducklings that were exposed to AFB_1_ due to its anti-oxidant capabilities [41]. Also, AFB_1_ was capable of augmenting oxidative stress marker, MDA, in the kidneys and blood. In contrast, they also observed that AFB_1_ was capable of reducing the anti-oxidant capacities of markers, such as nonenzymatic (glutathione) and enzymatic (glutathione peroxidase, glutathione reductase, and glutathione-*S*-transferase) [39].

Interestingly, vitamin E was capable of neutralizing AFB_1_ levels and restoring the parameter values above nearly the control level (Table 2) [39]. It was established that vitamin E was capable of sustaining integrity of long-chain polyunsaturated fatty acids in the membranes of cells, resulting in the preservation of their signaling molecules that could be modified by oxidative stress [148]. Moreover, vitamin E was capable of ameliorating AFB_1_-stimulated lipid peroxidation in the testis of mice as a result of its higher enzymatic and nonenzymatic anti-oxidant capabilities (Table 2) [91].

Furthermore, vitamin E was capable of influencing signaling function in vascular smooth muscle cells, resulting in its role beyond the antioxidative function (Table 2) [149]. Additionally, vitamin E had precise blockade effect on protein kinase C and a gene like collagenase [150]. Similarly, vitamin E had stimulatory effects on one protein, phosphatase, and on other genes such as alpha-tropomyosin and connective tissue growth factor [150].

Notably, antischistosomal drug oltipraz was capable of decreasing the disease burden associated with AFB_1_ during preliminary cancer prevention bioassays in AFB_1_-exposed rats [151–153]. It was capable of substantial but inadequate reductions in quantities of hepatic AFB_1_-derived DNA adducts in these animals (Table 2). Also, the pharmacodynamic action of the medicine was suggestive of augmented detoxication of AFB1 (Table 2) [154,155]. Also, synthetic oleanane triterpenoid 1-[2-cyano-3-,12-dioxooleana-1,9(11)-dien-28-oylimidazole (CDDO-Im) was capable of blocking AFB1-stimulated tumorigenesis in the rat (Table 2) [151].

The actions of CDDO-Im were obvious in the reduction of the hepatic focal burden of the glutathione S-transferase placental form (GST-P positive foci) of preneoplastic lesions [156]. Interestingly, CDDO-Im was a potent stimulator of Keap1-NF-E2-related factor 2 (Nrf2) signaling, which was capable of triggering augmented conjugation of the 8,9-epoxide of AFB_1_ with glutathione via the action of glutathione *S*-transferases (GSTs), resulting in the reduction of DNA adducts formed from this ultimate carcinogenic electrophile [157,158].

Furthermore, studies established that the protection offered by CDDO-Im in this model was attained mainly via the interaction with signaling pathways facilitated by the transcription factor Nrf2 [156,158]. Also, hepatic secretion of Nrf2 target genes, such as aldo-keto reductase 7A1 and GSTs, which were implicated in AFB_1_ detoxication, was elevated after CDDO-Im administration [151]. Thus, CDDO-Im functions as a multifunctional agent with anti-inflammatory, antiproliferative, apoptotic, and cytoprotective activities, influenced multiple targets and pathways (Table 2) [159,160].

In our perspective, medications, such as esculin, selenium, gynandra extract, vitamins C and E, oltipraz, and CDDO-Im, which have anti-oxidant functions, are capable of modifying AFB_1_-stimulated oxidative stress, resulting in the relieving of resultant anomalies. Thus, these drugs have to be re-purposed for the treatment of AFB_1_-associated infertility. We also advocate clinical trials on these medications for treatment of AFB_1-_associated infertility. Thus far, future research on the treatment of AFB_1-_associated infertility in both males and females should focus on these medications, which are already in use and readily available.

## 10 Conclusion

AFB_1_ is able to distract the reproductive systems in both male and female animals. Also, AFB_1_ is capable of interfering with the functions of several endocrine glands via the disruption enzymes and their substrates that are liable for the synthesis of hormones. Additionally, AFB_1_ is capable of influencing the key genes in endocrine regulation in placental cells after being metabolized into aflatoxicol, resulting in fetal anomalies. Moreover, AFB_1_ is potentially teratogenic and it is responsible for the development of malformation in humans and animals. Thus, AFB_1_ is one of the crucial markers to investigate in couples with infertility.

## Abbreviations

AFB_1_
: aflatoxin B1
Bax: Bcl-2-associated X protein
Bcl2: B-cell lymphoma 2
CDDO-Im: 1-[2-cyano-3-,12-dioxooleana-1,9(11)-dien-28-oylimidazole
CYP450: cytochrome P450
DHT: 5α-dihydrotestosterone
FSH: Follicle-stimulating hormone
GST: glutathione S-transferase
LH: Luteinizing hormone
MDA: malondialdehyde
Nrf2: NF-E2-related factor 2
8-OHdG: 8-hydroxy-20- deoxyguanosine
P5CR: pyrroline-5-carboxylate reductase
P5CS: pyrroline-5-carboxylate synthase
ProDH: proline dehydrogenase
ROS: reactive oxygen species
siRNA: small interfering RNA
SOD: superoxide dismutase
StAR: steroidogenic acute regulatory
T-AOC: total antioxidant capacity
UV: ultraviolet
Vitamin C: ascorbic acid
WHO: World Health Organization
